# The recent diatom-based paleolimnology of Lake Michigan

**DOI:** 10.1007/s10933-025-00363-1

**Published:** 2025-06-03

**Authors:** Euan D. Reavie, Andrew J. Bramburger, Meijun Cai

**Affiliations:** 1 Natural Resources Research Institute, University of Minnesota, 5013 Miller Trunk Highway, Duluth, MN 55811 USA; 2 Watershed Hydrology and Ecology Research Division, Water Science and Technology Directorate, Environment and Climate Change Canada, Burlington, ON Canada

**Keywords:** Great Lakes, Water quality, Eutrophication, Climate change, Silica

## Abstract

Quantitative analysis of siliceous microfossils in a dated sediment core from Lake Michigan reveals the anthropogenic history of pelagic conditions from the last ~ 160 years. Sediments deposited before the twentieth century contained low diatom abundances comprising species associated with oligotrophic conditions. Diatom-assemblage reorganization in the early to mid-twentieth century resulted in an increase in diatom-model-inferred water-column-phosphorus concentrations associated with cultural eutrophication. In recent decades, better nutrient management and water-quality recovery drove a decline of high-nutrient indicating diatom taxa. The most recent two decades manifest the effects of the extensive dreissenid invasion (a continued reduction in diatom-accumulation rate) and likely atmospheric warming (the rise in summer-diatom taxa representing a longer summer stratification and ice-free period). Like many areas of the Great Lakes basin, Lake Michigan’s paleolimnological sequence reflects the widespread eutrophication of the twentieth century, followed by remediation and a modern condition affected by multiple stressors.

## Introduction

Lake Michigan is the second largest of the Laurentian Great Lakes by volume (4918 km^3^) and third largest by surface area (58,030 km^2^) and is hydrologically contiguous with Lake Huron. Lake Michigan receives water from several major tributaries including the Fox (Wisconsin), Grand (Michigan), Menominee (Michigan, Wisconsin), Milwaukee (Wisconsin), and Muskegon (Michigan) rivers and discharges through the Straits of Mackinac into Lake Huron. Smaller but additional drainage comes from Illinois and Indiana. Its 118,000-km^2^ drainage basin contains over 12 million residents, making it the most populous drainage of all the Great Lakes. Lake Michigan is a temperate dimictic lake with a maximum depth of 281 m and a century-long water-residence time.

With the expansion of human populations within the Lake Michigan basin came several impacts to ecological integrity and impairments to beneficial uses of the system. Notably, increased phosphorus (P) loading during the mid-twentieth century contributed to cultural eutrophication. Schelske and Stoermer (1971) documented long-term silica depletion in the epilimnion in response to eutrophication-induced diatom blooms during stratified periods. Nalepa et al. (1998) reported declines in benthic macroinvertebrates, including *Diporeia* in nearshore areas of southern Lake Michigan between 1980 and 1993. The continuation of this phenomenon through the 1990s was reported by Madenjian et al. (2002), who also reviewed changes in the fish community of the lake. Salmonid stocking changed the forage-fish communities (Evans 1986), which in turn affected top-down control of zooplankton (Evans and Jude 1986) and possibly even phytoplankton communities (Fahnenstiel and Scavia 1987).

Several measures implemented under the Great Lakes Water Quality Agreement in 1972 (GLWQA; IJC 1972), including improved wastewater treatment and phasing out of high-P detergents, served to reduce point-source P loadings to the Great Lakes. Following actions prescribed by the GLWQA, P loading to the Great Lakes was largely reduced beginning in the 1970s, having a notable effect on reducing primary production (Dolan and Chapra 2012; Dove and Chapra 2015).

Widespread establishment of the zebra mussel (*Dreissena polymorpha* [Pallas]) in the 1990s was a primary driver of shifts in the macrobenthos community of the lake. More recent changes in pelagic physical, chemical, and biological properties have been largely attributed to heavy infestation by the profundal, invasive quagga mussel (*Dreissena rostriformis bugensis* Andrusov) (Hecky et al. 2004; Mosley and Bootsma 2015; Rowe et al. 2015; Vanderploeg et al. 2010). After colonizing the lake there were pronounced changes in the profundal benthic community (Nalepa et al. 2009) and the collapse of the spring phytoplankton bloom (Vanderploeg et al. 2010). Recent declines in chlorophyll have been greatest during spring mixing (Fahnenstiel et al. 2010), while declines in productivity have not been recognized during summer stratification (Reavie et al. 2014a) when stratification prevents filter-feeding mussels access to the photic zone. Further, changes in climate may also be reorganizing the primary producer community due to changes in lake physics that have led to stronger stratification, longer summers, and shorter and less-developed ice periods (Reavie et al. 2017; Warner and Lesht 2015).

Phytoplankton-community structure in the Great Lakes is a topic of considerable interest because these algae comprise an important component at the base of the food web that has been influenced by cultural eutrophication (Schelske and Stoermer 1971), invasive species (Vanderploeg et al. 2010), and climate change (Bramburger et al. 2017; Reavie et al. 2017). Biological surveillance programs have included phytoplankton-monitoring initiatives targeted at evaluating system-wide responses to stressors and management actions. Monitoring of modern phytoplankton communities through USEPA’s Great Lakes Biology Monitoring Program (GLBMP) has yielded considerable insight into the overall function of the Great Lakes algal community, as well as system responses to a variety of stressors (Bramburger and Reavie 2016; Reavie and Barbiero 2013; Reavie et al. 2014a). Despite the relatively long record of data generated by the GLBMP, the recent phytoplankton ecology has not been fully evaluated within a historical context (i.e. since European colonization).

Diatoms (Bacillariophyceae) are ubiquitously distributed in aquatic habitats and exhibit high-fidelity responses to environmental stressors. The silica frustules of diatoms are generally well preserved in sedimentary records and constitute an ideal means of inferring historical limnological conditions (Dixit et al. 1992). Reavie et al. (2017) demonstrated that the planktonic diatom communities of all five Great Lakes have become increasingly dominated in the last few decades by taxa considered characteristic of summertime assemblages, specifically taxa within *Cyclotella* sensu lato, and suggested that these changes were the consequence of anthropogenic climate change. Similarly, Bramburger et al. (2017) showed that diatom-cell size, both within taxa and across communities, has decreased on a basin-wide scale in response to stressors associated with warmer water columns. However, assemblage mean cell sizes in Lake Michigan showed no change through time, although species’ mean cell sizes increased over the past century and larger-celled taxa have been extirpated from the lake at disproportionately high frequencies during recent decades (Bramburger et al. 2020), alluding to a complex interaction between climatic and other stressors including dreissenid mussels.

In this study, we integrated the diatom paleolimnological record of Lake Michigan with human settlement in the Lake Michigan drainage basin to address outstanding questions around Lake Michigan’s phytoplankton history. In particular, we were interested in describing an updated long-term history since previous paleolimnological work. The latest comparable study by Stoermer et al. (1990) was based on a core collected in 1983, hence we aimed to provide a three-decade update to the fossil history of the lake. We broadly hypothesized that shifts in the diatom community were concurrent with changes in land use and water-quality management within the basin. We further anticipated that recent changes in diatom-community composition are representative of broader reorganization of the phytoplankton community in the wake of the dreissenid mussel proliferation within the lake, among other stressors.

## Materials and methods

### Sediment-core sampling

**Fig. 1:**
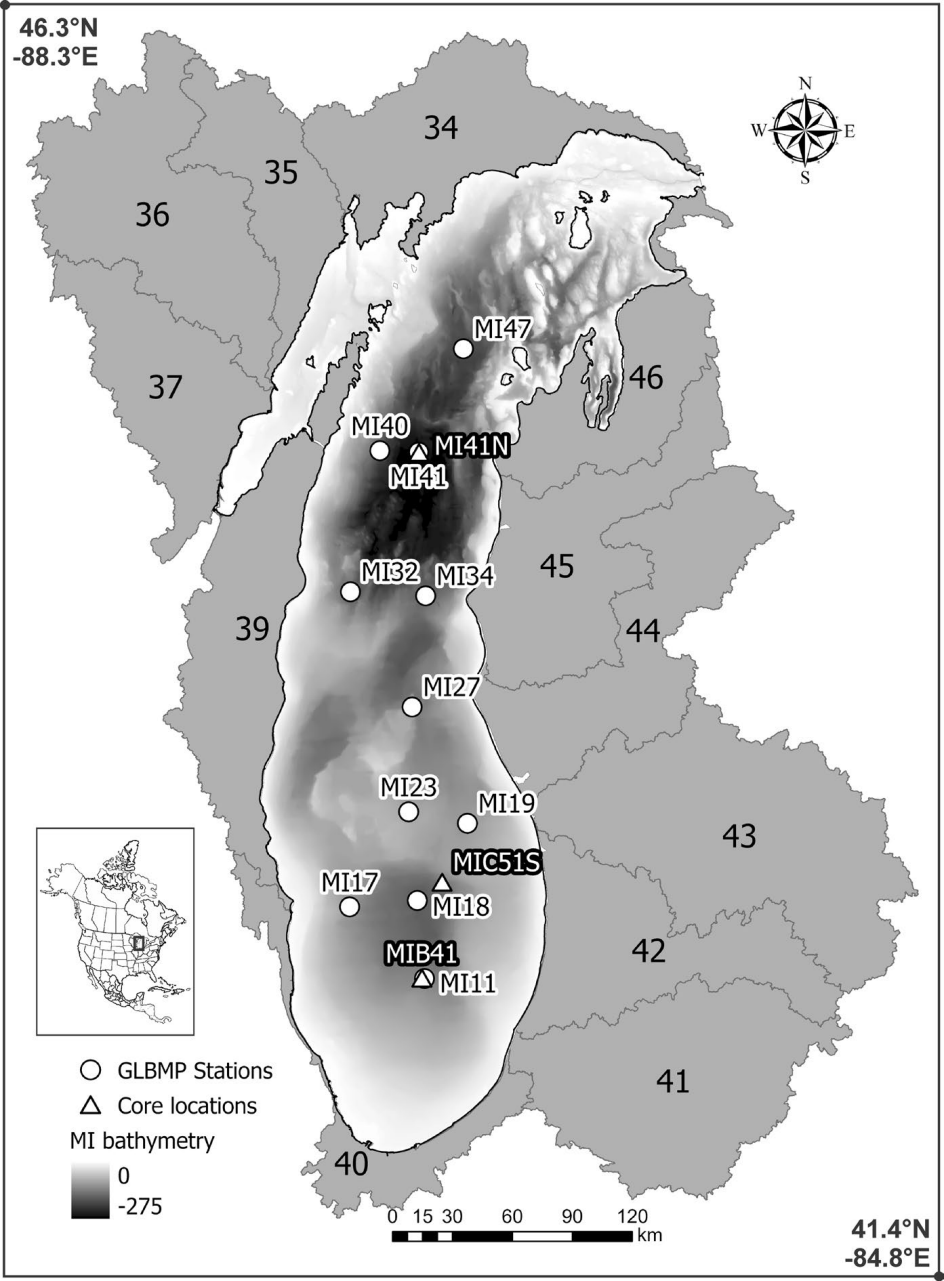
Lake Michigan with sediment-core locations marked as triangles. Circles mark USEPA Great Lakes National Program Office sample locations for the GLBMP. Core location MI41 N is the focus of this study. Sub-watersheds 34–37 and 39–46 for Lake Michigan were characterized for stressor data, as detailed by Reavie et al. (2018)

We selected coring sites based on known patterns of sediment accumulation in the lake (Cahill 1981). Two sites were sampled, one from the northern basin, also known as the Algoma Basin (April 2013, Research Vessel *Lake Guardian*, lat. 44.74°N, long. − 86.72°E, depth 240 m), and two from the southern basin (November 2009, Research Vessel *Blue Heron*, lat. 42.81°N, long. − 86.84°E, depth 127 m; lat. 42.39°N, long. − 87.02°E, depth 118 m) (Fig. 1). The northern site is near EPA-GLNPO sample station MI41 (core location MI41 N; Fig. 1) and is adjacent to a historical site where a core was collected in 1983 for paleolimnological analysis (Stoermer et al. 1990). The southern locations are within the convergent gyre (Kerfoot et al. 2008) that likely collects sediments from urban centers such as Chicago (Illinois). Sediments were collected using an Ocean Instruments model 750 box corer (30 cm × 30 cm × 90 cm). Sediment cores were extruded at 0.25-cm intervals for the first 20 cm, then at 0.5 cm from 20–30 cm, and 1-cm intervals to the bottom of the core. Unfortunately, it was later found that diatom preservation was poor in the southern cores so they are no longer considered in this article, though geochemical results from that core are included elsewhere in Great Lakes basin-wide analyses (Granmo et al. 2020; Reavie et al. 2021). We assume silica recycling is a major reason for poor preservation in the southern cores, but we do not know why there would be spatial differences. We recommend exploration of this phenomenon and do not discuss it further in this paper.

### Sediment dating

Subsamples from core intervals from core MI41 N were freeze-dried and analyzed for excess ^210^Pb activity to determine sediment age and accumulation rates based on methods presented by Appleby (2001). ^210^Pb was measured at 17 depth intervals in each core through its granddaughter product ^210^Po, with ^209^Po added as an internal yield tracer. Polonium isotopes were dissolved from 0.5–1.0 g dry sediment at 550 °C following pretreatment with concentrated hydrochloric acid and evaporated onto silver planchets (modified from Eakins and Morrison 1978). Activity was measured for as long as 45 days with ion-implanted surface-barrier detectors and an Ortec alpha spectroscopy system. Supported ^210^Pb was estimated from the asymptotic activity at depth (the mean of the lowermost samples in a core) and unsupported ^210^Pb was calculated by subtracting supported activity from total activity in upper levels. Dates and sedimentation rates were determined according to the constant rate of supply model (Appleby and Oldfield 1978) with confidence intervals calculated by first-order error analysis of counting uncertainty (Binford 1990). Lake Michigan cores had exponentially declining ^210^Pb profiles indicating typical isotopic decay with time and reliable accumulation with minimal disturbance and no discontinuities. Errors associated with dates ranged from 1–2 yr in the most recent two decades to ± 10–20 yr around ca. 1850. Detailed dating results for these Lake Michigan cores are provided in the supplementary materials of Granmo et al. (2020), and so are not discussed further in this manuscript.

### Diatom processing

Diatom valves were separated from the sediment organic matrix using standard techniques (Battarbee et al. 2001). Wet samples were digested in a beaker with a solution of 20 mL concentrated nitric acid diluted to 130 mL with deionized water. Digestion was accelerated by placing the samples on a hotplate at 100 °C until 20 mL remained. Then, 25 mL hydrogen peroxide (30% solution) was added using a catalyst of potassium dichromate and again heated until 10–15 mL remained. Digested slurries were rinsed several times in deionized water by iteratively spinning down samples at 2000 rpm and aspirating until slurries reached a neutral pH (approximately eight rinse cycles). Diatom slurries were mounted onto slides for light microscopy. Coverslips were prepared using the quantitative Battarbee et al. (2001) method, preparing two slides per sample interval by adhesion with Naphrax^®^ mountant.

Diatoms were identified and enumerated using an Olympus BH-2 microscope at 1000 × magnification with oil immersion. At least 400 diatom valves were counted per slide. The dimensions (length, width, diameter, and girdle depth) of the first ten encountered valves of each taxon were measured to determine average valve dimensions in each sediment interval (Reavie et al. 2010) for subsequent biovolume calculations. Identification of diatom taxa was based on in-house photographic records and plates (which have since been published by Reavie (2020, 2022, 2023), Reavie and Kireta (2015), and Reavie and Andresen (2020), and publications by Krammer and Lange Bertalot (1986–1991), Patrick and Reimer (1966, 1975). Assessment of slides for siliceous chrysophyte remains (stomatocysts, scales) was performed, but specimens were too rare to be useful in further analyses.

### Historical stressor data

Spatial and temporal land-use patterns for watershed population, agricultural area, and forested area were collected from historical records. A database was populated with information from various internet and GIS resources, which were tracked to provide metadata and documentation for each value (Reavie et al. 2018). Transcribed records combined a date, the land use in question (human population, agriculture, forests), the level of the land use (people, acres), and the spatial distribution of the land use. The level of each land use was summarized within 12 sub-watersheds covering the Lake Michigan basin. For this analysis, historical land-use data were generated for all 12 sub-watersheds combined to be used alongside concurrent fossil indicators from the sedimentary record.

### Data analysis

Hierarchical clustering of diatom-sample assemblages constrained by depth was applied using Euclidian distances to help determine where major composition shifts in diatom species occurred (R v4.4.1; R Core Team 2024) with the *rioja* (v1.0–6) package (Juggins 2023). A “broken-stick” analysis using the *bstick* function in *rioja* assisted in determining the minimum number of significant clusters (Bennett 1996). Rare taxa, defined as being < 3.0% of valve relative abundance in all samples counted, were removed from this and subsequent analyses.

Using *rioja,* weighted averaging (WA) calibration and regression were used to infer past total phosphorus (TP) water concentrations based on the environmental optima of diatom species. The modern training set used in this application was developed by Reavie et al. (2014b) and tested by Reavie and Juggins (2011). The training set consists of phytoplankton samples and concurrent TP measurements collected in 2007 to 2011 during the months of April and August. The latest model data are available from the authors.

To confirm the importance of TP as a likely driver of patterns in past diatom assemblages, Pearson correlation analysis examined the relationship between diatom-inferred TP (DITP) and the fossil diatom-assemblage scores represented as axis 1 of a principal component analysis (PCA; all ordinations were calculated using the R package *vegan* v2.6–6.1; Oksanen et al. 2024). A significant correlation suggests that the model data suitably reconstruct DITP for the core. PCA scores were based on log-transformed % species data with rare species removed (> 1% in at least three samples). Then, redundancy analysis (RDA; Blanchet et al. 2014) of samples constrained to DITP was used to derive a fossil diatom-TP relationship via the variance explained by RDA axis 1. The first axis-sample scores of the RDA were regressed against an unconstrained PCA of the same data to get λ_R_/λ_P_, which provided an assessment of how strongly TP (estimated as DITP) was related to patterns in the diatom assemblages (Reavie et al. 2014b). This ratio expresses the proportion of variation explained by DITP as a fraction of the maximum explainable variance in the sedimentary diatom samples.

A canonical correspondence analysis (CCA) constrained to TP was further used to evaluate the reliability of DITP values. Because the first axis in such an analysis represents the TP gradient, the residual distance of diatom-assemblage scores to the TP axis provides a measure to assess lack-of-fit to TP. CCA determined what were extreme residual distances from the TP axis on the basis of model data. Core-sample assemblages were then run passively in CCA and positioned, by means of transition formulae (Ter Braak 1990), with respect to the TP axis. Fossil samples with residual distances greater than the 95% confidence limits of the model-training set were considered to have poor fit to TP, thereby indicating DITP results would be unreliable.

## Results

### Land-use history

**Fig. 2:**
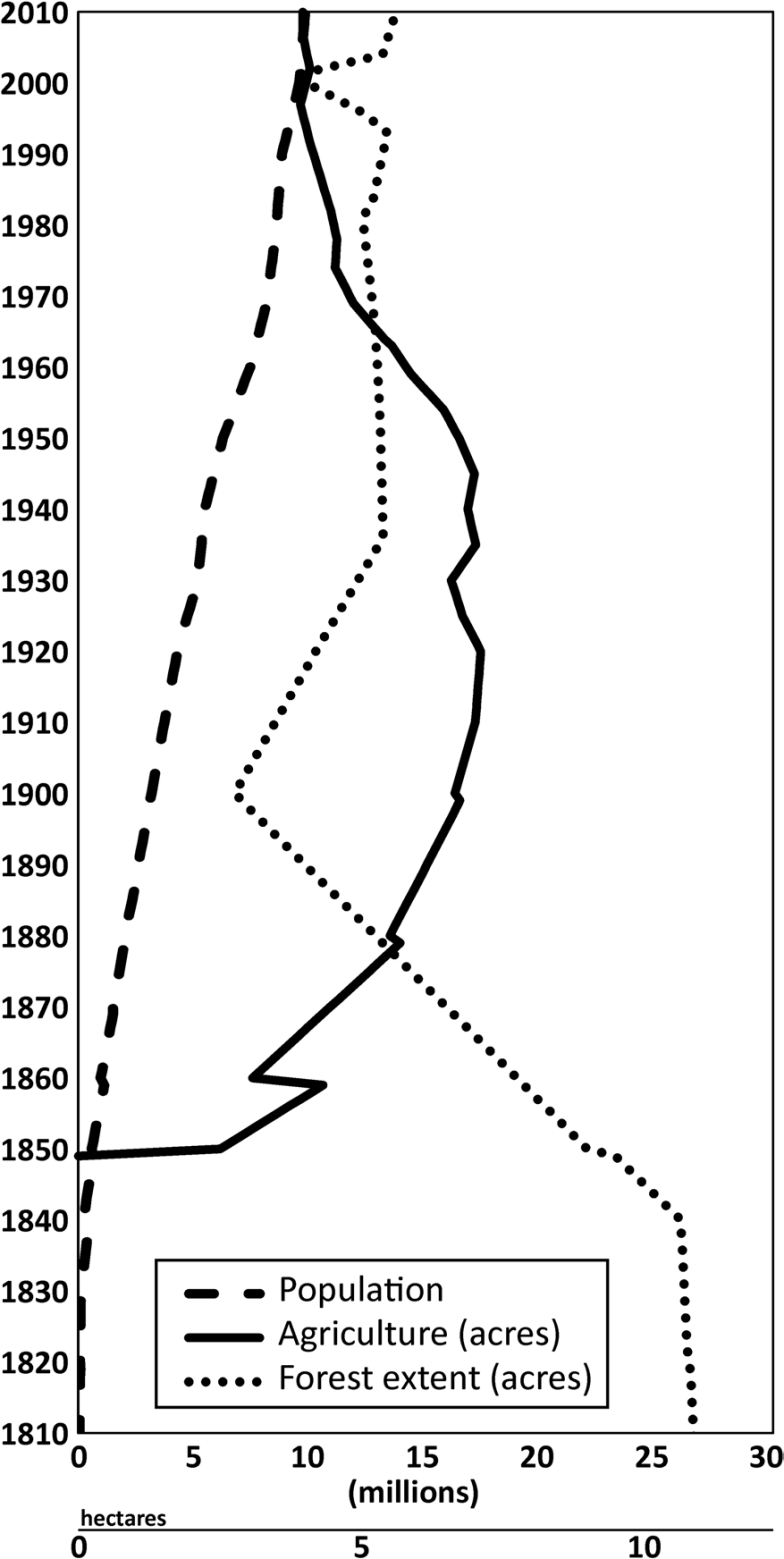
Historic anthropogenic stressor (human populations, agricultural acreage (one acre = 0.4 ha), and forested acreage) data from the watershed of Lake Michigan. Data were compiled from the Great Lakes historical stressor database (Reavie et al. 2018)

The start of population growth, deforestation and agricultural development began in the mid-1800s in Lake Michigan’s watershed (Fig. 2). This change in anthropogenic development is accentuated by a sudden growth in agriculture around 1850 and a gradual loss of approximately 20 million acres (8.1 million ha) of forest between 1840 and 1900. Human population increased gradually throughout the recent history represented in this study, reaching 3 million around 1900. Agriculture remained highest around 17 million acres (6.9 million ha) in the early half of the twentieth century, after which it declined to ~ 10 million acres (4.0 million ha) around the turn of the twenty-first century. The early twentieth century experienced forest regrowth from 7 to 13 million acres (2.8 to 5.3 million ha), after which it remained fairly stable up to present day. Throughout the twentieth century to present day, population continued to increase gradually from 3 to almost 10 million, with the last recorded data reflecting 2010 numbers.

### Diatoms

**Fig. 3:**
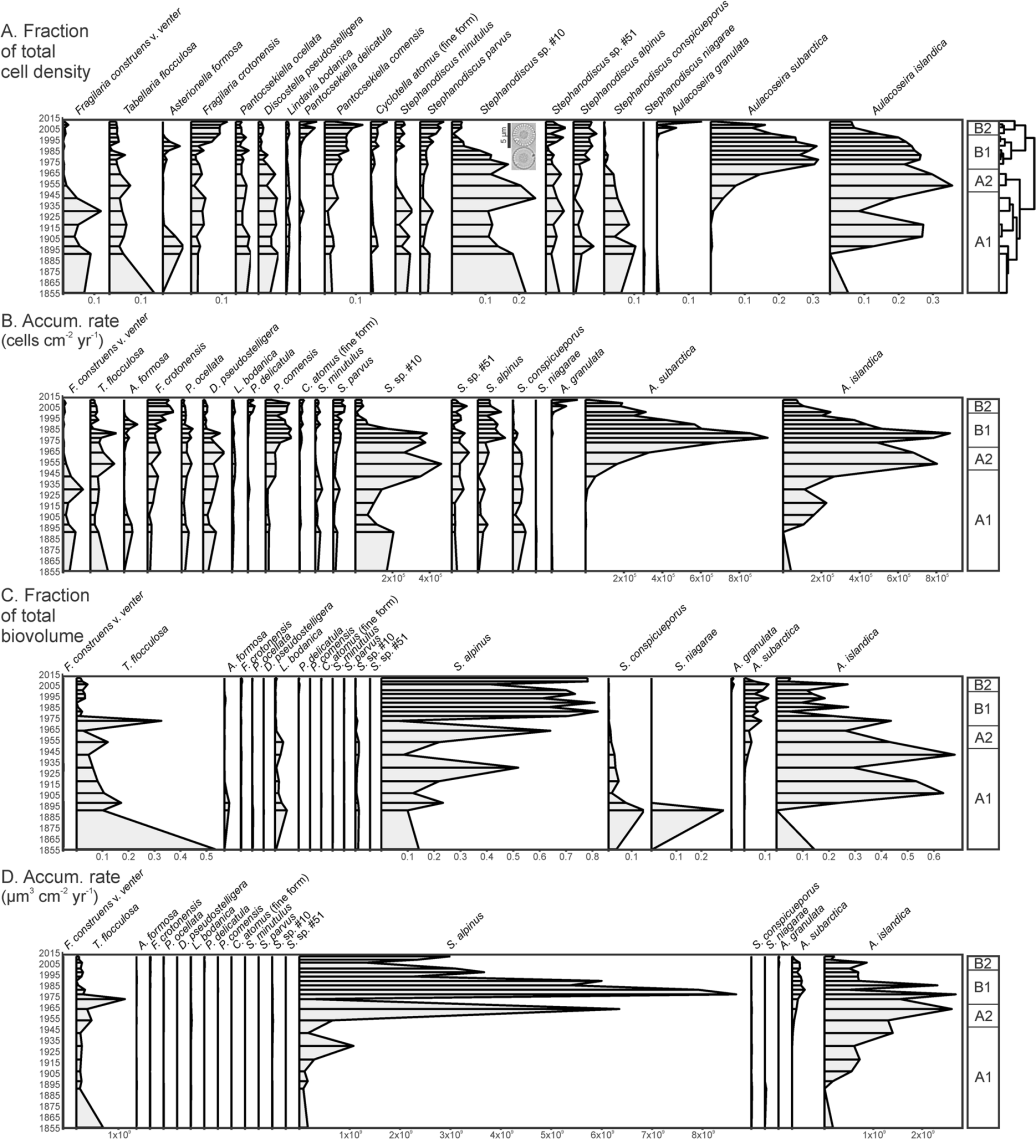
Stratigraphies of common (> 5% sample representation in any of the data types) diatoms from the Lake Michigan core MI41 N. Significant shifts in diatom composition (as identified by cluster analysis of the relative cell abundance, shown upper right) are marked as zones (A1, A2, B1, B2). The four profile sets represent the following properties of the taxa observed in the fossil assemblages: **A** fraction of cells of each taxon, **B** diatom-cell-accumulation rate, **C** fraction of each taxon’s biovolume, and **D** biovolumetric accumulation rate

Based on the relative abundance of diatom valves in sediment samples, two major temporal zones were identified: pre- and post-1970s, named zones A and B (Fig. 3).

Zone A. The earlier Zone A is further broken into a lower zone (A1) containing a pre-impact assemblage dominated by benthic taxa (*Staurosira construens* v. *venter* (Ehrenb.) P.B.Ham.) and several stephanodiscoid taxa (*Stephanodiscus conspicueporus* Stoerm., Håk. & Ther., *Stephanodiscus* sp. #10, *Stephanodiscus minutulus* Håk.), *Tabellaria flocculosa* (Roth) Knud. (represented by the so-called IIIP planktonic strain [Koppen 1975]) and *Lindavia ocellata* (Pant.) Nakov, Guillory, M.L.Julius, E.C.Ther. & A.J.Alverson. (*Stephanodiscus* sp. #10 is a small, concentrically undulate diatom that may be a species of *Cyclostephanos*, and is currently undergoing further taxonomic assessment. An example micrograph inset is provided in Fig. 3A.) Zone A1 transitions into Zone A2 around 1950, as these basal taxa started to decline in relative abundance to be partly replaced by filamentous, planktonic diatoms, largely *Aulacoseira islandica* (O.Müll.) Simonsen and *Aulacoseira subarctica* (O.Müll.) E.Y.Haw. Compared to upper portions of the core, Zone A1’s diatom-accumulation rate (0.6–1.3 million cells cm^−2^ yr^−1^) is relatively low in terms of cell and biovolume-accumulation rates, indicating that diatom production was much lower during this period. In Zone A2, total diatom-accumulation rates increased to ~ 2.3 million cells cm^−2^ yr^−1^.

Zone B. The upper zone in the core begins around 1970 and marks higher relative abundance of *Aulacoseira* as well as the continued decline in stephanodiscoid taxa present in Zone A. Zone B1 (~ 1970 through ~ 2000) was dominated by *A. islandica* and *A. subarctica* and included increasing proportions of *S. minutulus, Stephanodiscus parvus* Stoerm. & Håk.,* Stephanodiscus alpinus* Hust. in Hub.-Pest.*, Fragilaria crotonensis* Kitton and *Pantocsekiella comensis* (Grunow) K.T.Kiss & E.Ács. As assemblages transitioned into the uppermost Zone B2 (~ 2000 through 2013) these taxa continued to increase, and *Aulacoseira* also declined to be replaced by *Pantocsekiella delicatula* (Hustedt) K.T.Kiss & E.Ács, *Cyclotella atomus* “fine form,” and *Aulacoseira granulata* (Ehrenb.) Simonsen. Accumulation rates of diatom cells (up to 3.3 million cells cm^−2^ yr^−1^) and overall biovolume (up to 13.3 billion µm^3^ cm^−2^ yr^−1^) became much higher in Zone B. Cell accumulation was dominated by *A. subarctica* and *A. islandica*, while biovolume accumulation was dominated by the large-celled *S. alpinus*. Accumulation rates of both cells and biovolume declined in Zone B2.

Overall, considering the diatom microfossils as proportional and absolute abundance, and as cell and biovolume-accumulation rates, illustrates the extreme variation in importance of the various taxa to past diatom production. For instance, though *S. alpinus* was never more than 10% of the relative abundance, because of its large cell size it comprised up to 80% of the diatom biovolume since the 1950s. *Aulacoseira islandica* was also a major volumetric component of diatom production. This contrasts with the cyclotelloids and remaining stephanodiscoids, which were frequently abundant in terms of diatom-cell numbers but were much less important in terms of total production.

### Diatom-inferred total phosphorus (DI-TP)

Model testing criteria indicated the Great Lakes training-set model (Reavie et al. 2014b) is a suitable tool for reliably reconstructing TP from fossil diatom assemblages in Lake Michigan. Species overlap between fossil assemblages and communities that comprise the model ranged from 94 to 100%. The regression of DITP against PCA axis 1 scores of the diatom assemblages had a significant (*P* < 0.001) correlation of 0.83. Fossil assemblage scores (−0.19 to 0.80) fell well within the model 95% confidence interval of the CCA TP axis scores (−1.46 to 1.94), indicating good fit-to-TP of the fossil assemblages for DITP reconstruction. The ratio of λ_R_ (the variance in sedimentary diatom assemblages captured by the first RDA axis constrained to DI-TP) to λ_P_ (the variance explained by the first axis of an unconstrained diatom PCA) was 0.86. Because this value is close to 1, downcore assemblages were probably strongly related to changes in TP (Juggins et al. 2013).

**Fig. 4:**
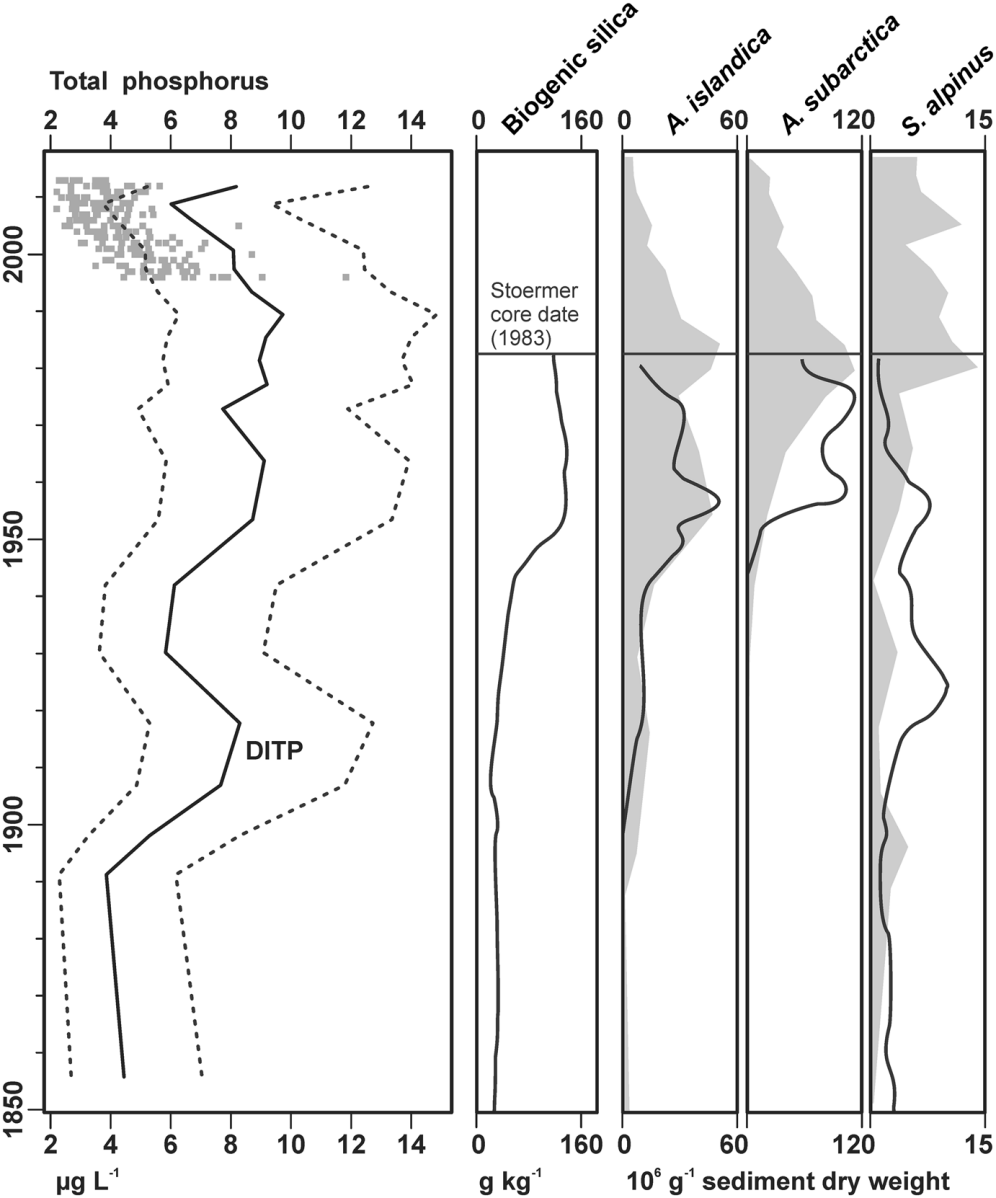
Diatom-inferred TP data (left panel) plotted as a solid line with the 95% model confidence interval as dotted lines. Gray dots mark measured total phosphorus data collected as part of the USEPA’s GLBMP (Fig. 1). Data from Stoermer et al.’s (1990) paleolimnological assessment (sediment concentrations of biogenic silica and the three biovolumetrically most important fossil diatoms) are presented in the four right-hand panels as solid lines. Light-gray shadows of the three taxa from the current study (accumulation rates from Fig. 3) are included for comparison

Quantitative reconstruction of water column TP (Fig. 4) indicates that cultural eutrophication began in the early twentieth century. Though concentrations were variable, prevailing DITP was approximately 4 µg L^−1^ prior to the twentieth century and increased to approximately 9 µg L^−1^ in the 1970s. Since then, DITP trended downward to as low as 6 µg L^−1^ around 2010. Measured TP also reflected this recent downward trend, though concentrations were lower with recent measurements centering around 4 µg L^−1^ which approximates the lower model error of the DITP model. Older Lake Michigan TP data summarized by Dove and Chapra (2015) indicate values around 7–8 µg L^−1^ during the 1970s, in better agreement with our DITP values.

### Other data sources

Though this is the first publication of the full diatom dataset from Lake Michigan, data from contemporaneous paleolimnology studies on Lake Michigan are worth considering alongside the diatom profile. Sediment-accumulation rates increased from ~ 1850 through to ~ 1890, indicating early stages of Euro-American development in the lake’s watershed (Reavie et al. 2021). Lake Michigan’s paleorecords had consistently increasing values of the stable isotopes δ^13^C and δ^15^N, suggesting increasing productivity through most of the twentieth century that corresponded with higher proportions of total organic carbon (%TOC) and total organic nitrogen (%TON). Lake Michigan underwent substantial inputs of allochthonous sediment around the turn of the twentieth century following periods of catchment development. The rise in terrigenous materials in the sediment record peaked during 1910–1950.

Sediment chlorophyll-*a*, a surrogate for historical lake primary production trends, increased from the early twentieth century through the 1980s in the sediment core from the northern basin (Reavie et al. 2021). A gradual rise in C:N ratio during the same period suggested increasing terrestrial sources, though the ratio always remained below 10, indicating phytoplankton as the main source of sedimentary carbon (Meyers 2003). Chlorophyll-*a* further tracked lower concentration trends in the most recent decade corresponding with the spread of filter-feeding dreissenids (Vanderploeg et al. 2010).

Monitoring data from Lake Michigan (Supplement S3 in Reavie et al. (2025)) record the recent decline in phosphorus and increase in silica in the deep, isothermal layers of the lake. This increase in the ratio of silica to phosphorus (Si:P) corresponds in the fossil record with a relative and absolute decline in heavy, silica-rich diatoms (e.g. *Aulacoseira*) and a relative increase in cyclotelloid taxa and *F. crotonensis*.

Though data from the previous paleolimnology work by Stoermer et al. (1990) were not available, we used approximated line traces of their stratigraphic profiles to compare with our new profiles (Fig. 4). Comparison with previous work indicates good correspondence between their profile of biogenic silica and the increase in large, volumetrically dominant diatom taxa (*A. islandica*, *A. subarctica*, *S. alpinus*). Taxonomy has changed since their 1990 publication; they identified *A. islandica* as *Melosira islandica* and we assume their *Melosira italica* corresponds with our identification of *A. subarctica*. Though there are some notable variations between old and new profiles for *A. subarctica* and *S. alpinus*, these taxa increased in concord with biogenic silica from ~ 1950 through Stoermer et al.’s core date of 1983.

## Discussion

This work provides approximately three decades of new paleoecological results from Lake Michigan since previous work by Stoermer et al. (1990). The last ~ 150 years of subfossil accumulation in the sediment record describe the twentieth century period of anthropogenic eutrophication, subsequent nutrient remediation resulting from management, and very recent changes associated with reengineering of the lake from a significant dreissenid invasion and rapid atmospheric warming, which has been verified from concurrent phytoplankton monitoring (Reavie et al. 2025).

The pre-impact taxon *Staurosira construens* v. *venter* (also known as *Staurosira venter* (Ehrenb.) Cleve & Möll.) is a known benthic taxon found commonly in nearshore environments of Lake Michigan (Reavie and Kireta 2015), typically on sand and mud substrates. It is rarely observed in Great Lakes phytoplankton collections (note the lack of inclusion with common taxa in the phytoplankton study by Reavie et al. (2014a)). All other common taxa in the core are phytoplanktonic, so the decline of this taxon in the mid-twentieth century reflects the increased dominance of phytoplankton via eutrophication. Further, the concurrent decline in accumulation rates for this taxon suggests it was being progressively outcompeted in coastal regions; rather, an unchanging accumulation rate would indicate this taxon was consistently productive and being replaced in relative abundance by pelagic taxa, but this was not the case.

The numerically important taxa early in Zone A tend to reflect species that are characteristic of oligotrophic lakes. *Tabellaria flocculosa* is a ubiquitous, oligotrophic (Reavie et al. 2014b) to mesotrophic (Reavie and Kireta 2015) species in the Great Lakes. Though stephanodiscoid taxa are generally considered tolerant of higher nutrient conditions, the early taxa *Stephanodiscus* sp. #10 and *S. conspicueporus*, which are known to be abundant during the winter-spring diatom bloom throughout the Great Lakes, have relatively low optima for phosphorus (Reavie et al. 2014b), which explains much of the oligotrophic signal inferred by the DITP model in the lower portions of the core. Further, the early cyclotelloids *Discostella pseudostelligera* (Hust.) Houk and Klee and *L. ocellata* have similarly low phosphorus optima, which is typical for species in this group (Cremer and Wagner 2003).

Although major deforestation and agricultural development began in the mid-nineteenth century, which would have increased the potential for nutrient flux to Lake Michigan and likely contributed to the increase in sediment load, responses of the phytoplankton in the form of cultural eutrophication did not notably begin until the turn of the twentieth century. Efficiently produced synthetic fertilizers were developed in the early twentieth century, with extensive use by 1940 (Russel and Williams 1977), so in addition to a lag time in lake impacts, this richer form of fertilizer may have played a role in this early period of eutrophication.

*Aulacoseira islandica* is a well-known fossil indicator of increasing nutrients in the Great Lakes, particularly in Lake Superior (Shaw Chraïbi et al. 2014) and Lake Erie (Sgro and Reavie 2018), and its high abundance in the fossil record contributed much of the inferred DITP signal during the twentieth century. It is also associated with winter conditions, growing under ice and blooming during early spring, so the recent lessening of winter intensity may be contributing to the recent decline in *A. islandica*. There is probably a complementary phenomenon where dreissenid filter-feeding during winter-spring overturn is a potent loss term for these phytoplankton. *Aulacoseira subarctica* is considered a mesotrophic taxon (Reavie et al. 2014b), and its increasing presence later in the twentieth century further reflects nutrient enrichment. The mean cell sizes in Lake Michigan fossil records noted by Bramburger et al. (2017) are very likely associated with the increase in these large diatom species including the robust *S. alpinus*, which is also considered an indicator of mesotrophy (Reavie et al. 2014b).

Schelske and Stoermer (1971) and Stoermer et al. (1990) advanced and studied the hypothesis that reduction of bioavailable silica was a major factor during early cultural eutrophication in Lake Michigan. This assumed that increased P loading resulted in increased diatom production that would consume water-column silica and that the siliceous remains of the diatoms would be archived in the sedimentary record. The hydraulic renewal time for Lake Michigan is long (~ 100 yr), so an increase in silica storage may result in decreased available silica in the water column. This would limit diatom growth in summer, favoring non-siliceous phytoplankton. In Stoermer et al.’s (1990) study it was revealed that progressive eutrophication of Lake Michigan in the early twentieth century likely culminated in silica limitation during summer stratification after the mid-1960s, and indeed biogenic silica increased substantially in the upper portions of their Lake Michigan core. Though we did not analyze for biogenic silica, accumulation rates of diatom biovolume (largely due to robust, heavily silicified taxa such as *A. islandica* and *S. alpinus*) during the late twentieth century up until Stoermer et al.’s coring date of 1983 directly corresponds with their quantitative silica observations. Since then, the combination of recovery following nutrient management and the reduction of pelagic biomass by dreissenids has substantially reduced silica storage in the sediments, as is evident by the loss of large-diatom biovolume in the uppermost sediments (Fig. 3). This has likely driven the return of higher silica concentrations in the water column, based on spring and summer pelagic collections since the 1990s (Evans et al. 2011; Mida et al. 2010). Further, the associated rise in Si:P, which is generally considered a nutrient shunting effect of the quagga mussel invasion (Hecky et al. 2004; Li et al. 2021), may be supporting the reorganization of diatom communities. In growth-culture experiments of Lake Michigan natural algal communities at varying Si:P (Kilham 1986), *F. crotonensis* had a relatively high affinity for high Si:P, which concurs with our observation of increasing *F. crotonensis* cell abundance in upper sediment intervals. While this taxon is a small portion of the spring algal biovolume in pelagic Lake Michigan, in terms of propagules (e.g., proportion of cells mL^−1^) it appears to be an important indicator of stoichiometric changes.

The large differences among biovolume representation in the diatom taxa illustrate that a few taxa (*A. islandica, S. alpinus, T. flocculosa*) have made up most of the diatom biomass in Lake Michigan. This is important to note as these biovolume data help to track historical production trends, though taxa such as the cyclotelloids, which comprise a very small portion of the total diatoms in the fossil record, are well-known, critical indicators of lake condition.

The recent rise in taxa from the diatom clade *Cyclotella *sensu lato is a phenomenon that has been recognized throughout the Laurentian Great Lakes (Reavie et al. 2017). Several possible drivers showing trends during the same period include the sum of nitrates and nitrites (increasing), total phosphorus (decreasing), atmospheric temperatures (increasing), winter-ice coverage (%, decreasing), and silica (increasing). The dominant cyclotelloid taxa increasing near the top of the core (*P. comensis, P. delicatula*) are known summer species, so it is not surprising that they have relatively high temperature optima (Reavie et al. 2014b). However, regression analyses between cyclotelloid abundance and temperature have been inconclusive on this phenomenon (Kireta and Saros 2019). We propose that temperature is not a direct driver of cyclotelloid abundance (in agreement with Anderson 2000), and instead a lengthened summer period of stratification and shorter ice period (illustrated by Ozersky et al. (2021)) allows for a longer period of reproduction for these species in the late months of every year, resulting in an increasing annual period of frustule deposition to the fossil record. Though such a scenario requires additional sampling to confirm this mechanism, it may explain relative increases in cyclotelloids in recent sediments.

The recent appearance of *A. granulata* is interesting because it is typically associated with higher summer-nutrient concentrations (Reavie et al. 2014b; Stoermer et al. 1981), while a decline in surface nutrient concentrations has been verified (Barbiero et al. 2018). *Aulacoseira granulata* also has a particularly high optimum for temperature (Reavie et al. 2014b), so the well-known warming surface waters may be a factor in its abundance. This taxon was not noted in Stoermer et al.’s (1990) core from 1983, indicating that its presence is a new observation for Lake Michigan. Recent dreissenid activity is having an ecosystem-scale effect on nutrient cycling and depositional patterns in Lake Michigan (Ozersky et al. 2015; Pilcher et al. 2017), so it is a fair assumption that *A. granulata* is gaining some relative advantage under the reengineered lake system. We further note that, as recent as 2022, *A. granulata* is rarely observed in Lake Michigan phytoplankton collections in April and August, suggesting it occurs during an unsampled time of year. Given its high temperature optimum, mid-winter presence seems unlikely and instead it may occur during ephemeral periods of extreme warming and stratification. Mechanisms for this relationship remain speculative.

Our DITP model (Reavie et al. 2014b) for the pelagic Great Lakes was determined to be suitable for use in Lake Michigan, and it provided a satisfactory inference of historical eutrophication and oligotrophication trends. The comparison with modern phosphorus measurements (Fig. 4) indicates that the model may overestimate water-column-phosphorus concentrations, but we caution that the diatoms in the fossil record represent year-round diatom-frustule accumulation and, therefore, may be a better indicator of prevailing, year-round water quality. The DITP model is based on snapshot April and August measurements, so refined taxon calibration to phosphorus may benefit from the addition of water quality and phytoplankton collections from other times of year, such as winter.

## Conclusions

This fossil assessment of Lake Michigan provided a clear record of increasing human effects throughout the twentieth century, with nutrient recovery occurring in more recent decades, followed by multi-stressor drivers of the modern phytoplankton conditions. The combined effects of multiple stressors like invasive species and atmospheric warming demonstrate the challenges facing Lake Michigan and highlight the need to continue improving our understanding of mechanisms affecting primary production. Ongoing research to fill knowledge gaps about lake conditions—e.g., winter-phytoplankton dynamics, species responses to climate-related shifts—will provide crucial interpretations on lake trajectory, in light of recent changes and observations associated with Lake Michigan’s pelagic primary producers.

## Data Availability

Diatom data from Lake Michigan are available as a supplement to Reavie & Cai (2019), and the diatom-based model data are available as a supplement to Reavie et al. (2014a).
